# “If I Accomplish Nothing Else but Hope, That Will be Enough”: The Ethics and Morality of Market-Based Hope in Illness Entrepreneurship

**DOI:** 10.1007/s10551-025-06230-3

**Published:** 2026-01-06

**Authors:** Susi Geiger, Olya Loza

**Affiliations:** University College Dublin, Belfield, Dublin 4, Ireland

**Keywords:** Ethics of hope, Ethics of care, Illness entrepreneurship, Markets for hope, Chronic illness, Moral markets, ME/CFS

## Abstract

How do entrepreneurs embody and enact hope as a motor for ethical agency—agency that builds one’s own and others’ capacities and capabilities to overcome a painful present? This study puts hope at the center of theorizing around the activities of entrepreneurs who leverage it both as a way to sketch a new life for themselves and to sell it to others. Through the case of illness entrepreneurs—recovery coaches for those suffering from chronic illnesses—we observe the moral struggles involved when hope becomes framed within the logic of the market. Recovery coaches at once embody hope (as formerly sick people who have recovered), neatly package and sell it (as entrepreneurs), and manifest it (opening up new spaces of possibility for themselves). The work involved in balancing these threads requires constant realignment of the entrepreneurs’ own hopes for a new life and their care for others. We show how, through these moral struggles, hope opens up new spaces of possibility and imbues people—both entrepreneurs and their clients—with the agency to move toward those new horizons. Conceptualizing the complex entanglements of hope, care, and ethical agency, our study represents a foray into recent calls for assembling and theorizing the ‘architecture of hope’ in organization and entrepreneurship studies. We also discuss whether this entrepreneurship, balancing hope and care to enable ethical agency, opens up new ways of understanding morality and agency in market contexts.

## Introduction

Hope has always been a motor of entrepreneurial practices. It particularly fuels the work of marginal, less well-resourced, or otherwise ‘untypical’ entrepreneurial subjectivities (Dimov, Johnson & Sørensen, 2025). From those engaging in ‘emancipatory entrepreneuring’ to free themselves from societal shackles (Karlsson, 2025) and ‘barefoot entrepreneurs’ scraping a living through entrepreneurship (Doussard et al., 2023) to those who practice entrepreneuring as part of their creative professions (Bolton et al., 2012), entrepreneurial striving is often permeated with hope for a less precarious, more self-directed, or more meaningful life (Michaelson, 2021). As Dimov et al. (2025) have recently explained, the ethics of entrepreneurial hope is a deeply agentic one: to shape a future that overcomes the problems of the past, at an individual and a societal level. Yet, in moving toward a better future for themselves and others, entrepreneurs may encounter a major tension: while they seek to overcome current oppressive economic or social conditions for themselves, they often channel this hope through existing market structures, which may be oppressive or harmful to others (Martin et al., 2024).

A recent body of literature has started to interrogate how, through their participation in the market system, entrepreneurs – and particularly those on the margins of that system—perpetuate the very conditions that have placed them in less-than-hopeful situations to start with. Indeed, if hope, for these entrepreneurs, translates into “the act of making a claim on the future for themselves and their loved ones” (Ringel, 2021, p. 885), this claim is frequently tied to adopting and adapting to the logic of the market (Boltanski & Thévenot, 2006). Such hope can be ‘cruel’, to speak with Lauren Berlant (2012): it can place people in the metaphorical waiting room of an economic system that will never fully accept them or their ways of entrepreneuring. What’s more, such hope feeds what Petersen and Wilkinson (2015) call the ‘magic system’ that is kept alive by neoliberal markets: the belief that one can reach a better life only in and through the market. Bound within a market logic, hope not only becomes a motor for participation in an often exploitative system, but it frequently also becomes a product that is sold on to others, modeling practices that are ultimately unlikely to foster ‘ethical agency,’ which Johansson and Wickström (2023) define as ‘caring with others.’ Yet, despite business ethics scholars’ deep engagement with the feminist ethics of care (e.g., Alacovska & Bissonnette, 2021; Geiger et al., 2024), the intertwinements between care, hope, and ethical agency in market contexts are yet to be fully explored.

Answering a recent call to business and organization scholars to engage with the ‘architecture of hope’ (Hudson et al., 2025), we ask: How do entrepreneurs embody and enact hope as a motor for ethical agency—agency that builds one’s own and others’ capacities and capabilities to overcome a painful present? We interrogate the relationship between entrepreneurship and hope in markets where hope is packaged as a form of care by individuals who seek to transcend their own situation through entrepreneuring. This type of entrepreneurship has emerged in various lifestyle, wellness, and healthcare markets, where those with embodied experience coach others in areas from weight loss to yoga and mindfulness to addiction therapies and holistic medicines (Moriarty, 2024; O’Neill, 2024). This economic space is broad and multifaceted—the wellness market alone was estimated at between US$2 and 6 trillion globally in 2023, with figures pointing to a compound annual growth rate of 5–6% (McKinsey 2025; Global Wellness Institute, 2024). While some areas of healthcare such as pharmaceuticals or medical services are tightly regulated, others—including prevention and rehabilitation services—are subject to light-touch or no regulation, which opens the doors to entrepreneurial entry. In these types of services, ‘illness’ and ‘wellness’ entrepreneurship are often indistinguishable from one another: while ‘illness’ entrepreneurs may define themselves as focusing on prevention of disease and rehabilitation of patients, wellness entrepreneurs provide services that enable people to incorporate health-positive behaviors into their lives (Global Wellness Institute, 2019). It is precisely because of its many gray aspects that this space presents an ideal territory to map the complicated entanglements of hope, care, and ethical agency in entrepreneurship and to uncover the complex moral struggles that result when entrepreneurs reconcile their own hopeful practice of entrepreneuring with the market-based individualization they create.

We study an area of illness entrepreneurship where hope, care, and market logics are particularly deeply entangled: recovery coaching for people living with chronic energy-limiting illnesses, particularly Myalgic encephalomyelitis (commonly known as chronic fatigue syndrome or ME/CFS), and Long Covid. Both are chronic conditions that affect many of the body’s systems and manifest through a myriad of physical and cognitive symptoms, above all an overwhelming fatigue that is not improved by rest (Bansal et al., 2022; Jason et al., 2024; NICE, 2021). Both are also “pervaded by medical, social and political uncertainty” (Dumit, 2006, p. 577). For both illnesses, it continues to be difficult to obtain a diagnosis, and securing one rarely guarantees access to treatment—if treatments exist at all (Deale & Wessely, 2001; Macpherson et al., 2022). In the absence of certainty, hope—for a diagnosis, for treatment, for research breakthroughs, for access to benefits, for improvement, and ultimately for recovery—plays a crucial role in many people’s experiences of these illnesses. No one embodies hope more than those who were able to recover from these illnesses and have since started to offer their services as ‘recovery coaches’ to others with chronic energy-limiting conditions. Recovery coaches at once embody hope (as formerly sick people who have recovered), attempt to package and sell it (as entrepreneurs), and manifest it (as people forging a new life for themselves after the disruptions wrought by lengthy illness). We use the term ‘recovery coaches’ as most of our research participants self-identified as coaches. In addition, we frame their work as ‘illness entrepreneurship’ to take account of the patient-facing work that these coaches are engaged in, in contrast (but, as we will see, with some overlaps) to ‘wellness’ or ‘lifestyle’ entrepreneurs (Glover et al., 2024; Ivanycheva et al., 2024; O’Neill, 2024).

Our findings map the different roles that hope plays in fuelling the care practices of these entrepreneurs and the tensions that framing hope through a market logic may present. Observing the moral struggles that result when these former sufferers reconcile themselves with marketizing and selling hope to others, even in the face of significant uncertainty around recovery trajectories, our findings demonstrate a complex ethics of hope: hope is entangled in various ways with entrepreneurs’ experiences of suffering and vulnerability, the care they seek to take of fellow sufferers, and their own economic prospects. Confronting and resolving these moral struggles allows these entrepreneurs to find an always-precarious balance between opening up spaces of possibility for themselves and for others—a balancing act that we describe as engendering ‘ethical agency,’ which we define as agency that builds one’s own and others’ capacities and capabilities to overcome a painful present (see also Johansson & Wickström, 2023). We conjecture whether seeking and striking the balance between self- and other-agency and using hope as a guardrail to lead to new spaces of possibility ultimately makes for a type of ‘moral’ market that has so far been neglected by the ‘moral markets’ literature (Geiger & Gross, 2018; Balsiger, 2021; Caruana, Glozer, & Eckhardt 2020; Casasnovas et al., 2025). Yet, in the final count, it becomes apparent that market-based hope remains individualized hope that is unlikely to lead to systemic change—in contrast with, for instance, hope being expressed in an emancipatory collective practice such as patient activism (Vidolov, Geiger & Stendahl, 2023).

We draw two conceptual contributions from our deep engagement with hope entrepreneurship: first, we offer a new way of conceptualizing ‘morality’ in markets, particularly in those markets where shared pain, suffering, or vulnerability provide the foundation for market encounters; and second, we trace the first contours of an ethics of hope, analyzing how care, hope, and ethical agency work together to move people out of painful presents and toward new spaces of possibility. Our findings hold relevance for many other organizational settings that display similarly complex entanglements between hope and the market, in medical markets as well as for lifestyle, wellness, and craft entrepreneurship (e.g., Ivanycheva et al., 2024; MacDonald & Gavura, 2016; Moriarty, 2024).

## Hope and the Logic of the Market

Conceptually, our inquiry sits within a burgeoning space of research on hope in non-mainstream entrepreneurship, which has gained in importance as the work-lives of many have become increasingly precarious, non-linear, and therefore often involuntarily more entrepreneurial. Endrissat and Lüthy (2025, p. 723) have called for “greater attention to how … different forms of hope are cultivated and affect entrepreneurial processes” in these marginal spaces. They call for closer examination of what they call ‘critical hope’—hope that is couched within a market logic yet also illuminates ways to transcend its own complicity with the market. Hope, in such contexts, becomes a moral practice that balances between ‘complicity’ in often oppressive systems and ethical agency as a ‘passport’ for a better life (Endrissat & Lüthy, 2025; Hage, 2003). Whether it is marginalized creative workers in Eastern Europe who build themselves “islands of hope amidst a sea of despair” (Alacovska, 2019, p. 1120) or museum curators in Russia who tease out the degrees of freedom they can gain while conforming with the regime (Endrissat & Lüthy, 2025), hope for these entrepreneurs represents a “temporal, quotidian and ethical practice of coping” (Alacovska, 2019, p. 1124) with less-than-hopeful economic and life contexts.

Yet, despite isolated empirical advances in business ethics and a strong line of conceptual research in social theory, hope remains an elusive entity (Ringel, 2021). Theorized in many different forms, hope has been described as a principle (Bloch, 1959), a method (Miyazaki, 2006), a Christian ethics of action (McCarty, 2020; Moltmann, 2012), a politics of change (Zournazi, 2002), and a moral project (Mattingly, 2010; see also Pedersen, 2012). In recent organizational scholarship, hope is considered as an affective and relational phenomenon, a force that circulates between bodies and can ‘stick’ to them (Ahmed, 2004). Ahmed’s work teaches us not to ask what an affect such as hope ‘is,’ but rather to study what ‘work’ it does: what effects it has on people’s lives and on the ways they relate to each other. Inquiries into the ‘work’ of hope often focus on those spaces where hope is situated “in the context of different forms of suffering” (Anderson, 2006, p. 744), and they trace how collectives transcend such suffering through hopeful, future-oriented agency. Indeed, where suffering is related to experienced injustices, hope can become a driving force for political organizing, resistance or change. Vidolov et al. (2023) for instance examined the interplay between pain and hope in patient communities and saw hope as an affective glue that keeps patient collectives pushing for change in spaces where conventional market mechanisms prove inadequate. Similarly, Wright (2023) articulates how hope helps those involved in land resistance movements remain in the struggle against economic injustices and to understand their lives differently, and McCarty (2020) recalls the case of a community group in Tijuana, Mexico, who built simple homes for migrants swept up there by the global currents of capitalism.

In many of these contexts of resistance and struggle, it is hope’s particular temporality that renders it agentic. Hope emerges through embodied relationships in the present yet displays a commitment to the possibility of a better future—an “openness and insistence on possibility” (Wright, 2023, p. 1505), or, with Ernst Bloch, a movement toward the ‘not yet’ (Vidolov et al., 2023). Kleist and Jansen (2016) summarize hope’s peculiar temporality as a ‘future-in-the-present,’ and Lear (2008) envisions ‘radical’ hope as directed toward a future that cannot even be fully imagined, a “good for which those who have the hope as yet lack the appropriate concepts with which to understand it” (p. 103). This forward-looking, agentic temporality, which opens up new spaces of possibility and moves people toward them, is also built into the Christian ethics of hope (Elliott, 2017; McCarty, 2020; Moltmann, 2012). Social psychologists too have noted that hope as an ethical practice enables individuals to develop their own goals, points toward pathways to fulfill these goals, and fosters agency for individuals to embark on these pathways (Corn & Feldman, 2024; Snyder, 2002). In contexts of political organizing, this agentic, future-oriented impulse allows hope to become a “material force with performative effects” (Anderson & Holden, 2008, p. 144), continuously bringing new relations, new agencies, and new realities into being.

Much of the literature mentioned so far considers cases where hope is framed in emancipatory terms, often related to overcoming oppressive economic or political systems. Yet, in those cases where hope manifests through entrepreneuring, it can also be a propelling force *toward* market attachments, even at the risk of perpetuating larger structures of economic injustice. A special issue of the journal Environment and Planning D has been dedicated to this inquiry (see Ringel, 2021), demonstrating, for instance, how entrepreneurs ranging from homeless people in Paris to Ghanian weavers to Nepalese migrants and Brazilian street vendors engage in diverse ‘hopeful’ practices in order to gain access to global capitalism’s market structures. Self-agency, in these cases of entrepreneuring, is related to hope for betterment in one’s own life, not necessarily to ‘caring with’ (Tronto, 2013) or empowering other marginalized individuals or groups. Indeed, as Anderson (2006, p. 747) highlights: “there are numerous occasions when the enactment of hope catalyses relations of injustice” rather than alleviating them. Marxist scholars Thompson and Žižek (2014) see the ‘privatization of hope’ as a pervasive feature of late-stage capitalism, contrasting more emancipatory forms of hope (as conceptualized in the work of Ernst Bloch or Latin-American liberation theologists) from hope that is subservient to a market logic, with the latter making people vulnerable to exploitation and despair.

This intertwinement between hope and self/other-agency becomes even more complicated in those spaces where hope becomes the good that is packaged and sold to others, as it is done in many contexts of care. Harnessing hope in these marketized contexts may turn out to be highly uncaring; it can engender new dependencies and the “dangers of evangelical beliefs” (Norrito, 2024, p. 1). For instance, the care technology of ovarian tissue freezing for female cancer patients can foster hope in a future where women’s fertility is restored (Bach & Kroløkke, 2020), yet as a ‘hope technology’ it can also be extremely uncaring when patients are sold a baffling number of add-ons to their fertility treatments at great financial and emotional cost (Perrotta & Hamper, 2021). Prasad (2015), similarly, charts the ‘journeys of hope’ to India that patients undertake to avail of highly speculative private stem cell therapy treatment—a market in which care, clients’ vulnerabilities and the ‘magical thinking’ that market-based hope entices lie in particularly close vicinity. Researchers have started to pay attention to how the discourse of hope in contemporary market-based care settings is often accompanied by an undertone of individual responsibilization, with hope becoming an “exercise of choice, personal control, and empowerment” (Petersen & Wilkinson, 2015, p. 115). Hope that is “both generative of and generated by market interests,” according to Eaves et al., (2016, p. 37), is individualized and often cruel hope, which ‘glues’ the individual to the market rather than representing an agentic force for change.

Curiously, while a burgeoning literature has emerged around the notion of ‘moral’ or ‘moralized’ markets, this research focuses either on the ethical attitudes and motivations of buyers and sellers or on how to evaluate and improve the ‘goodness’ of the products or services sold (Geiger & Gross, 2018; Balsiger, 2021; Caruana, Glozer, & Eckhardt 2020; Casasnovas et al., 2025). This literature has little to say about how ethical agency is fostered or impeded by market logics and structures. This seems a vital issue to examine, particularly in the context of care markets and other forms of ‘caring’ market relations (Geiger & Loza, 2024).

Overall, existing inquiries present a highly complex picture of market-based hope as an affective and relational practice that precariously balances between present and future, between cruel optimism and emancipatory agency, and between settings that exploit people’s deepest vulnerabilities and those that are embedded in an ethics of care (Chatzidakis et al., 2025; Geiger et al., 2024; Vidolov et al., 2023). Asking how entrepreneurs embody and enact hope as a motor for ethical agency, we seek answers to the following questions: Can hope retain its agentic impetus in market contexts of care, rather than getting people stuck in relations of dependency and suffering? How do entrepreneurs of hope reconcile *self-*agency with care for others to enact ‘ethical agency’? What moral struggles result from this tension, and how do entrepreneurs resolve them? And finally, is there any evidence that these individual moral considerations may collectively lead to a more ‘moral’ market—a market that bestows people with agency rather than taking it away from them? Taking these insights into contemporary care contexts may help us understand if and how marketized hope can foster people’s agency to move toward new spaces of possibility, rather than closing them down through market-based magical thinking and cruel optimism.

## Methods

In seeking to establish how entrepreneurs can enact hope as a motor for ethical agency, we need to study up-close the spaces of possibility that are opened up or closed down in these market-based contexts. We explore these issues in the market for ME/CFS and Long Covid (henceforth simply ME/CFS) recovery services. This market is one in which both sellers and buyers are often desperate for hope: the entrepreneurs in this market are frequently former sufferers who had to contend with major biographical disruptions caused by the illness and who have to reinvent themselves. They draw on their experience—both of being ill and finding their way to recovery—to offer their services as recovery coaches to the wider ME/CFS, Long Covid, and chronic illness community. Importantly, they also typically develop their services in response to what they perceive as the failings of mainstream healthcare markets and the hopelessness that they experienced navigating these markets. They engage in entrepreneurship often as somewhat accidental entrepreneurs, having undergone major life and financial disruption through prolonged illness but rarely having planned for an entrepreneurial existence.

The topic and aims of this inquiry emerged from a broader study that sought to understand the lived experiences of people with chronic energy-limiting conditions. As part of this broader project, we conducted 46 narrative interviews (Pederson, 2013) with people who have (or have had) ME/CFS and/or Long Covid. We also carried out an art project with some of our research participants, which offered us a more visceral and ‘felt’ understanding of the illness experience than standard interviews convey.^1^ During these inquiries, hope (and, frequently, its absence) transpired as one of the major life themes of people living with ME/CFS. We also noticed that some former sufferers had started to pursue a new entrepreneurial livelihood through selling their own recovery experiences as a service. On the basis of these insights, the current paper is based on a more targeted empirical inquiry concerning the market for hope built in this space of suffering: extensive secondary data collection and 12 narrative interviews (ranging between 75 and 120 min) with 11 recovery coaches (one was interviewed twice) who were all former ME/CFS sufferers. Table 1 describes our participants’ details and aliases.

**Table 1:** Research participant details (pseudonymized)

	**Initials**	**Gender**	**Age**	**Location**	**Background**
1	MV	F	50–60	Spain	Recovered by using the Gupta Program^a^ and is now a coach with the program
2	LD	F	30–40	US	Trained as a coach and offers one-to-one coaching. Has a website where she blogs about living with/recovering from chronic energy-limiting conditions, as well as sharing interviews with other people who have recovered. With another coach, she has created a ‘programs guide’ that outlines the key differences between different recovery programs available
3	FU	F	50–60	Spain	Recovered by using the Gupta Program and is now a coach with the program
4	NT	M	20–30	Canada	Recovered with the help of his doctor, a psychiatrist, and has used the insights gleaned from his own recovery to, initially, offer short, free advice videos on YouTube and, eventually, create and launch his own group recovery program, which at the time of the interview cost $6,000 to join
5	DM	Other	50–60	US	Coach, energy practitioner, aroma therapy expert
6	PJ	F	40–50	UK	Started her own ‘fatigue coaching’ business where she mostly works one-on-one with clients
7	VR	F	50–60	Germany	Recovered by using the Gupta Program and is now a coach with the program
8	TA	F	30–40	US	Recovered on her own, through a combination of various methods and approaches; started by sharing her own recovery story on YouTube and now hosts the most popular recovery channel on the platform, where every week she interviews people who have recovered from ME/CFS and other chronic conditions about their recovery stories (she has posted over 400 videos and has more than 30 K subscribers)
9	AV	F	40–50	UK	Recovered through somatic healing. Offers one-to-one coaching sessions and hosts annual healing summits, which bring together medical professionals and researchers, but also holistic and somatic healing experts, trauma experts, natural healers, etc. Also hosts a podcast
10	BH	M	40–50	UK	A therapeutic coach who runs a fatigue clinic that largely focuses on nutrition and psychology; has written books about his ME/CFS recovery; hosts an online learning platform which offers conferences, courses and workshops on fatigue, trauma, anxiety, sleep and relationships; and offers training courses for prospective coaches
11	QT	F	30–40	US	Recovered through somatic healing and is offering one-to-one (and some group) coaching focusing on holistic, somatic and trauma healing

^a^The Gupta Program is a ‘train the trainers’ program focusing, according to its website, on brain retraining and neuroplasticity, in which several of the coaches in our study trained

Most of the coaches have only a small number of one-to-one clients, while some run group programs. Only one person has set up and operates what he calls a clinic—a physical premise—with over 1000 patients. Many ‘bricolage’ their work, supplementing private and group coaching with running private recovery support groups, making YouTube videos interviewing other people who have recovered, or publishing advice on managing the illness, often tied into promoting their recovery programs. As Table 1 illustrates, while several of our research participants were based either in the US or the UK, coaches operate from different countries and often have an international client base, as most offer their services online and in English.In all cases, clients have to pay for these services out-of-pocket; even in countries with public health insurance systems, recovery coaching for ME/CFS is typically not recognized as reimbursable medical treatments.^2^

In addition to the interviews, our secondary data collection efforts focused particularly on the digital sphere. Vidolov et al. (2023) have shown that this sphere is an important setting for the circulation of hope in patient communities, and indeed, many of the recovery coaches have an active digital presence with websites, YouTube channels, podcasts, online conference hosting, or blogs, which we scrutinized. Overall, we collected, read or watched, and analyzed three books and two documentaries; 21 articles in traditional media; 126 newsletters and blog posts by the recovery coaches we interviewed as well as by one coach we have not been able to interview; 17 episodes of a podcast by our research participant AG; 29 interview transcripts on LD’s website; a sample of 20 (out of more than 300) recovery stories on TA’s YouTube channel; and each of the coaches’ websites. These materials provided a crucial backdrop for our understanding of the coaches’ work, as well as a sense of how they want to be perceived and what they communicate to their prospective clients. They also gave us a sense of the scale of their commercial endeavors and provided an important opportunity to triangulate our interview insights.

The analysis that underlies our findings presented below was an emergent and iterative process. We started with ‘close readings’ (Ellerman, 2024) of our first few interviews and the secondary materials and had several meetings in which we discussed the themes and ideas that have struck us the most. From these individual and shared readings, we selected a series of possible areas of questioning for further inquiry, including the moral struggles emerging in the coaches’ narratives; their motivations in pursuing this work; their sense of self/purpose/identity; and the good they see themselves as doing. While continuing with our fieldwork, we surveyed a range of literatures around entrepreneurship, ethics of care, vulnerability, hope, and patient activism in search of ‘moments of resonance’ between concepts and data (D’Antone, Geiger & Fuschillo, 2025). In doing so, we found that this particular group of coaches sits oddly with many existing literatures: not quite wellness entrepreneurs, but not patient activists either; not explicitly manipulating their clients for profit, but not offering advice on managing illness as a free service to their community either—they sat somewhere at the intersection of many different market- and self-identities. Similarly, existing conceptualizations around conventional entrepreneurship often did not quite fit with the more craft-like, emergent, and improvised nature of the work undertaken by recovery coaches, which was reminiscent of creative workers (Alacovska, 2019). Exploring this literature prompted us to ask what kind of entrepreneurial existence they were building, and what its key organizing principles were. Finally, the ethics of care literature echoed some of the themes emerging in our empirical inquiry, particularly around shared vulnerability as a basis for relationality (Geiger et al., 2024; Johansson & Wickström, 2023) and around the balancing acts between self- and other-agency (Alacovska & Bissonnette, 2021). Yet, other aspects such as the strong future orientation of the coaches’ work and their moral struggles around agency resonated less strongly with this literature (though see Antoni, Reineke & Fotaki, 2023). Repeated readings of our initial thematic data analysis and further advances in our understanding of the illness through our art project, which we conducted in parallel, confirmed that while many of these insights were important, the main moral struggles of these entrepreneurs revolved around marketizing hope and how to make this hope foster ethical agency, which our participants described as balancing self- and other-care. As we will show, this ethics of hope ran through our data in different guises: the coaches’ hope for more life-sustaining work, their clients’ hope for recovery, and how this hope is both ‘sold’ and ‘given’ to others, a continuous balancing act that put significant pressure on the coaches. Below, we unpack our understanding of recovery coaches’ complex and multifaceted relationship to hope and the way it becomes an organizing principle for the market for recovery services.

## Findings

### Hope Against the Market: Leaving a Painful Present Behind

Recovery coaches in our study often openly positioned themselves *in opposition to* conventional healthcare markets, which they portrayed as uncaring, exploitative, and agency-destroying. All of the coaches in this study identified themselves as ‘coaches’ or ‘mentors’ and offered one-to-one sessions and/or structured group programs aimed at improving and managing symptoms and, in some cases, therapy-adjacent emotional support. None of the coaches we interviewed had or claimed to have medical education or credentials, and many underscored that their work was necessary precisely because of the failures of mainstream medical markets. As one of the coaches in the study explained, people with ME/CFS.

…have tried getting help before from so many different medical professionals that didn’t help them, or straight up lied to them and said, “Yes, here take this supplement and this treatment, it is $5000, $10000, it will help you.” They pay that money and then they are still sick. So that trust is kind of broken. (NT)

Many coaches in our study spoke of their own sense of hopelessness faced with their diagnosis and subsequent encounters with mainstream healthcare practitioners: as QT put it, “I did feel very misunderstood and cast aside by doctors, and I saw close to 50 practitioners.” Against this help- and hopelessness emanating from mainstream markets, all of our coaches saw it as their mission to offer hope to people suffering from ME/CFS that there *can be* a future—that one day they too might heal. They emphasized time and again how their own experience of living with the illness—and how difficult it was to find help—prompted them to want to offer the help and support they sorely missed during their own illness. BH, for example, who runs a busy fatigue clinic, said that he “set up the clinic that I had wanted to exist when I had been ill” to “support the community that it serves.” Similarly, NT said: “When I was sick, I told myself if I ever got better, I wanted to make a program online that helps people go through this.” PJ and NT both expressed a similar sentiment, of seeking to ‘do good’ and ‘give back’ by sharing hope:

I wanted to give people what I needed back, you know, 13 years ago that wasn’t there. That is literally at the core of everything is that I wanted to give people what I needed that wasn’t there. (PJ)

AV likewise based her entrepreneuring on “32 years of experience with it”—‘it’ being both the illness and the labyrinthian array of medical therapies he had availed of over those years, adding not only that he’d spent tens of thousands of pounds trying to get better but also that “doctors have been fairly crap most of the way through.” Thus, coaches’ entrepreneuring was deeply motivated not only by their illness experiences but also their experiences of a medical market that had let them down and that continued to let other people down. The care that they sought to enact as part of their services was to extricate fellow sufferers from this painful present—it was thus from the start cast as fostering others’ agency. It is perhaps all the more striking in the face of this staunch rejection of exploitative market structures that they nonetheless decided to package and sell their own services through the market, rather than, say, offering it up as a free service to the patient community. This requires further unpacking.

### Embodying Hope: Opening up a Space of Possibility for Others

Against a distinctly careless market that often actively took hope away from people and left them stuck, coaches set out to imbue hope to fellow patients. This happened mainly by embodying hope for recovery through their own journeys, and thereby opening up a space of possibility for others. As one of the coaches in our study put it in her newsletter:

If you find yourself not feeling ok right now, I hope my weekly newsletters are inspiring you to start believing you *will* be ok one day too! (TA, newsletter from 26 June 2024)

There was a sense among many of the coaches we interviewed that recovery was quite rare, and so if they found a way to achieve it, they had a moral duty to share that with others. This was FV’s motivation: “this is what I want to spread. It [the recovery regime] worked for me, it could work for many.” MV echoed this sentiment:

Yeah, when I was totally fine for me it was like a miracle and I was also like, ‘Come on, this works’. It is a chronic illness, there are so many people suffering, people have to know. (MV)

Likewise, TA shared her initial motivation in starting her YouTube channel as acting as a beacon of hope for others:

I’m going to put my story out there and I am going to tell everyone what works for me. Not that it is going to work for everyone but I thought this worked so well for me it is going to work for other people. (TA)

Many spoke about how ‘inspired’ (LD) they themselves had felt as sufferers when they came across recovery stories online. Though there is now no shortage of such stories, at the time when many of the coaches in our study were trying to recover from ME/CFS, they were few and far apart: as TA explains, “I didn’t think that there were other people […] that had recovered, but there were so many. The conception out there, or the misconception is that nobody recovers.” In light of this scarcity of hopeful stories, coaches sought to “be part of the wave of change” (LD) that opens up spaces of possibility and encourages sufferers not to lose hope:

I want to educate people what this is, but do it in a hopeful lens [sic] I think we have got to give our inner child hope. And there is a kid in all of us, like 1 to 92. And older people need hope as well. I think we are all real kids inside, we need that hope. (LD)

TA similarly stated that “if I accomplish nothing else but hope, that will be enough,” a phrase we adopted for this paper’s title as it powerfully signals how embodying hope worked as a fundamental motivation for entrepreneurs in this context. The phrase also expresses how hope works as ‘more than’ a marketized affect, functioning as a binding fabric between people experienced with and experiencing shared pain (Scarry, 1985). Similar to some community addiction recovery programs such as Alcoholics Anonymous (AA) or those that have emerged for opioid addiction, the shared embodied experience of suffering and vulnerability stands firmly at the center of interactions between those recovering and those who already have recovered, and stories and embodiments of overcoming this suffering are the main source for shared hopefulness (Beddoe & Beddoe, 2019). In a recent newsletter, TA communicated the following:

Every one of us who’s come out of the ME/CFS or Long Covid maze has suffered A LOT. And it changes you. When you get better, you really understand and care about others who are still sick. Sure, some of us move on, but others just can’t shake off the feeling of needing to do something about it. That’s where health coaches come in. They’re our community’s unsung heroes, stepping up where the traditional medical system falls short. I would have recovered so much faster if I’d had someone who’d been through it all to guide me. […] And with millions of people still facing these conditions, we need more health coaches! What do you think? It’s of course wise to be cautious about whom you reach out to for help, but I think we should cherish and celebrate each and every one of these incredible people making a difference for our community. (TA, 9 April 2024)

Here, TA articulates how hope has a corralling and binding effect, much in line with previous studies on patients who use hope as a motor for political activity (Vidolov et al., 2023). The obvious difference between Vidolov, Geiger and Stendahl’s patient activists and our recovery coaches is the latters’ decision to make a business out of giving hope to their fellow sufferers. In the most extreme case, this involved charging people up to US$6,000 for a recovery course. When probing these decisions in interviews, it transpired that how the coaches experienced and navigated the resulting tensions also related to hope—in this instance, their own hope for a life, and livelihood, after recovery, as we will highlight next.

### Enacting Hope Through Self-Agency: Nurturing One’s Own Space of Possibility

Hope manifested in our interviews with recovery coaches strongly not just in care for others but also in their own hopeful dispositions toward the future, particularly their vision of fleshing out a new way of living and working. There are commonalities here with those lifestyle or craft entrepreneurs who seek to forge alternative ways of being through their entrepreneurship (Alacovska, 2019; Ivanycheva et al., 2024). Similar to those entrepreneurs, all of our coaches were accidental entrepreneurs. Many would have never considered an entrepreneurial existence before their illness, most found starting on this path difficult, and the path itself felt virtually uncharted:

I can’t tell you how unsure I was that this was going to actually work. Because there was nothing like this. All I was going by was that I kind of needed it. I had been the person who, over the years, if one of my friends heard of somebody else who had been diagnosed with ME/CFS they would go, ‘Oh you need to talk to [PJ].’ …But I didn’t know that there would be a demand as far as people would pay for it. (PJ)

Underpinning their perseverance was the realization that their own recovery might represent a ‘good’ that could not only help others but that could also be marketized, even if it was unclear how to do so. Most of the coaches described the process of setting up their businesses as “pretty organic and intuitive” (QT); some had what they referred to as a loose business plan: “it was definitely just taking the next step, and sort of trying this and realizing I like this and there’s a need for it” (QT). PJ explained that she did not set out with a ‘clear vision’ but allowed the business to emerge dynamically as time went on:

Five years ago I didn’t sit there going so I am not going to promise to fix people, I am just going to help, it wasn’t that calculated. And like any good practitioner I am always prepared to refine and change and meet demand or what have you. (PJ)

Hoping that this pathway would eventually lead to success, coaches often started to piece together work across different platforms and ways of engaging prospective clients. QT, for example, started out by offering free coaching sessions to people she knew and took motivation and hope from the feedback she’d receive:

[Initially] I didn’t even have a website or anything. I found some of them [her clients] like, I think I sent out a mass email to friends and family, and just said, you know, I’m offering free life coaching or mind-body coaching sessions, and then I also, in some of the trainings I was in, they would have break out groups or small group discussions, and I would just say, I’m offering some free sessions. So it wasn’t like, I was, you know, marketing myself. It was kind of like that, and then it started spreading through word of mouth. (QT)

Bricolaging a new existence gave our coaches a strong sense of agency in moving away from their pre-illness livelihoods, which were often seen as causally contributing to their illness. Going back seemed impossible to all of them: they could no longer pursue the careers they had before they got sick, whether because the demands of those jobs were no longer sustainable in light of how their bodies respond to stress, because those jobs no longer aligned with their inner values, or because they had contributed to becoming sick in the first place. QT had considered returning to her career as a broadcast journalist but quickly decided against it:

it truly felt like that was a chapter that was gone, and I had, part of me had almost died, you know, and other parts have been reborn, and I was a different person. I didn’t want to do that, and it’s also a very stressful, dysfunctional career path. (QT)

Similarly, LD admitted: “I was so burnt out by my own job I didn’t want to do that again. That is what contributed to getting there, being out of alignment.” With this old, ‘out of alignment’ life impossible to return to, the prospect of recovery coaching opened up a new space of possibility where alignment may be found. ‘Achieving alignment’ was an often-repeated phrase and typically referenced alignment between what the illness journey had taught our interviewees and the values they now wanted to enact:

You know being at a bank was not aligning with my values. Even worse than working in a bank, I worked in marketing for a bank, and our job was all about tempting people to get loans and overdrafts and credit cards for the things that they couldn’t afford, which is just horrendous, isn’t it? […] as I got older, it felt less and less something that I wanted to do and that, coupled with my joy of being back and loving having my life back, it was more a sort of a ‘Ahh, those two things could actually align!’ (PJ)

Similar to other lifestyle entrepreneurs, the act of entrepreneuring through recovery coaching was thus a deeply agentic one: it became a way to pursue alignment between the kind of work they pursue, their values, and the life they want to live—a way of life that MV described as flow and that she was eager to model to others. Though maintaining this alignment was not always easy, as we will illustrate next.

### Ethical Agency: Balancing Self- And Other-Agency.

One of the persistent moral struggles our participants described was trying to help as many fellow sufferers while keeping themselves from overworking and relapsing. Achieving alignment was not only about living the values they wanted to put out into the world; it was also about finding a balance between the amount and type of work they do and their personal wellbeing. QT for instance emphasized how she continuously sought to balance her own wellbeing needs with helping her client base:

I think that, probably the biggest challenge now is managing the growth of the business, and how many people I can coach and teach, with my own mind-body needs, and finding balance in my lived work-life […] So I’m trying to find ways to operate in a compassionate way to myself and others, and be a bit more, you know, efficient, find more balance, all the while making it a business, you know, because it is how I support myself […] I’m looking at what serves this population of people and myself. (QT)

QT continues to explain that central to achieving alignment was to learn to manage her “intake of emotional information” by limiting how many people she sees each day:

I can see three people a day. I mean, I don’t know how therapists see like eight people a day, but my sessions often go a little longer, like 75 minutes, and I can see four a day, but I don’t feel I can hold the same presence. … And it’s challenging sometimes not to fall back into old habits of perfectionism and hard-driving behaviors, even though it’s for this good cause. So I think for me personally, that’s just the edge I’m playing with this year. (QT)

PJ similarly said she was ‘very disciplined’ about her personal boundaries and limits to her energy reserves: “I protect what I have got.” For her, this means that there was a ceiling to how many people she can support at any one time. That was not a straightforward calculation, however: “that is a constant battle for me mentally because I want to help more people” (PJ). She deployed several additional strategies to establish firm boundaries around her professional work and her personal life. Interacting with the reality of sufferers every day, she said, gives her.

…a reminder every single day about how awful these health challenges are, and I can tell you that I get something from that personally because it is a constant reminder of where I have been and what I have got back from, and the fact that I really want to stay here. (PJ)

Thus, for our coaches, building a recovery coaching business was a way through which they can move closer to embodying the values that the illness has taught them, serve the community they belong to, and earn a livelihood for themselves. Getting the balance right between self- and other-care meant that goals were continuously readjusted, depending on the energy people had available; the desire to help more people had to be constantly balanced with the threat of relapse. NT was the only coach in our study to explicitly articulate a different approach, saying he was prepared to ‘sacrifice’ parts of his life in the short run for achieving a future business horizon. Perhaps not very surprising, playing this ‘edge’ between self- and other-agency also colored how coaches packaged and sold hope as a market object.

### Moral Struggles Around Marketizing Hope

Making a living out of selling hope of recovery is an endeavor layered with complexity, with coaches negotiating many decisions on an ongoing basis. The newsletter by TA that we quoted above opened with the following statement:

The Internet is such a wild place and I’m (mostly) used to getting all sorts of comments from people on my videos and posts. The majority are so helpful and incredibly kind and I have learned a lot over the years from what people have taken the time to share.  But there are some that make me scratch my head. A big one is when people talk about health coaching like it’s just about selling things, and it’s confusing to me. (TA, 9 April 2024)

The confusion TA expresses is interesting. This statement (and similar ones in other coaches’ interviews) lays bare an underlying shame or guilt associated with ‘selling hope’: the guilt of asking sick people, who are often at the end of the rope physically, emotionally, and financially, to pay for another uncertain stab at recovery—another hope for a different life when so often before that hope had turned out to be no more than magical thinking.

A central decision related to the question of how to marketize hope was how to price their services. Many coaches in our study spoke about starting off by seeing clients for free and only charging for their services when they felt confident enough in what they were offering. This was particularly important for those who were piecing together their own programs rather than following an established treatment paradigm that would lend them a sense of legitimacy from the get-go. QT recounted how she realized the value of what she had to offer:

I just started seeing people without charging for quite a while, like almost a year, and then I sort of felt like, Wow! They’re really having shifts, and they’re getting a lot out of this, and I really have something to give. So I started charging very low rates. (QT)

She added that ‘slowly, over time’ she started charging people more—until reaching the point where her sessions are now priced at US$170:

People might think, Oh, she’s making a lot of money. But I’m actually barely supporting myself. Because, you know, I only see 3 people a day. (QT)

Recovery coaching, it appears, is not a very profitable enterprise. In part, its lack of profitability is because the emotional and time intensity of coaching create very tangible limits on how many clients can be seen in a given day. This was further complicated when coaches sought to be fair in what they charged their clients. LD admitted that “I haven’t really earned any money from what I’m doing” and PJ, who used to have a high-paying job, echoed her: “I earn enough, but I am certainly not doing this for the money.” PJ recognized that part of the reason she is able to pursue this work is the fact that she and her partner maintain a ‘pretty simple’ life and that as part of her new ‘alignment’ she is no longer preoccupied with wealth accumulation. Others spoke about their coaching work being enabled by family, inheritance, or (most often) their partner’s job, with LD admitting that the bills adding up from her coach training “is putting pressure on my marriage, too.”

Prices for coaching sessions, then, have to allow the coaches to meet their own basic expenses; as BH noted, “in terms of how we price the services, we just, we try not to lose money is basically the strategy.” The morality of their entrepreneuring plays strongly into these considerations, particularly in relation to widening access to the services offered:

Really, it is a challenging dynamic, to make a living at this career, and help people who don’t often have a lot of money. It is and sometimes it’s just – I don’t want to say it’s an inner conflict, but I very much care about helping people. And I do want to support myself, and I actually need to support myself, you know. So I actually just continued to live with my mom while I was doing all these trainings and launching my business because we’re really close, and it just enabled me to have a little more freedom to help people without having to charge rates that I would need to support myself where I live. (QT)

The moral struggle that QT describes in this quote was, at times, exacerbated by encountering pushback from patients with ME/CFS who expect that ex-sufferers would offer their services as part of supporting the community. This hints at a conflict between those who saw hope circulating as something that was communal and inimical to market logic and those who sold it to build a hopeful existence for themselves—or, in our conceptual terms, between self- and other-agency:

a big struggle that seems to be for me and everyone I talk to is that there is a lot of resentment from a lot of the people out there from us doing this because we are trying to get rich off of their suffering and everything we do should be for free. There is a lot of that feeling out there. Like if we were really good people we would never charge for anything ever because we know what they are going through. (TA)

The coaches knew from their own experience how difficult it could be for people with ME/CFS to afford recovery services—a lot of people with ME/CFS are forced to leave their jobs while spending significant sums of money in search for treatment. A distinct ethics of care emerges in this balancing act between self- and other-agency, nurtured from coaches’ own biographical experiences. VR for instance recalls that she spent over €2,000 per months on supplements at one point, taking around 100 pills every day. FU and PJ both reflected on how these biographical experiences influenced their entrepreneurial decisions:

If people don’t have money I have always taken in that many clients on next to nothing to show I care. I know that it’s so hard to get by. Because it’s awful. You don’t get anything with this disease. (FU)

…it was very important for me to be seen as wanting to help and, because as you said, people are usually in a financial pickle. They are either already in a financial difficulty or they are worried about being in one in the future because they don’t know how long, how it is going to go. And it doesn’t seem right to me to expect people to give me thousands and thousands of pounds in that situation. (PJ)

Almost all of the coaches we interviewed–with the exception of two, NT and BH—adopted a flexible approach to pricing their services, out of a duty of care. LD, for example, charges wealthier clients more and might charge other clients a bit less. Some of the coaches had created scholarship funds or offered cheaper group courses. MV outlined a range of strategies she uses to make sure she can help as many people as possible, while also making a living:

I charge what I have to charge and I earn money from that because it is my way of living. But I also get clients who cannot afford it and then I will work […] For example they pay me short sessions and I give them a one hour. I help them in crisis for free, things like that. […] to other ones I charge what I have to charge. (MV)

Similarly, DM worked with groups of people and offered grants and sliding scale pricing to some of her clients to maintain at least some of her work on a more affordable level, while also working with higher-paying clients that bring in more money.

Despite all their concern about the affordability of their programs, which illustrates coaches’ care for fellow sufferers, a surprising consensus emerged about the fact that no matter how little, it is important their clients pay *something*. This was typically couched as a way to ensure that the clients would remain committed to—in fact, many used the word ‘invested’ in—their recovery pathway. AV recounts that some of her clients go: “Oh! That’s quite a lot!” when she mentions her hourly rate but explains that “I want them to be a bit uncomfortable, and I want myself to be a bit uncomfortable, so I want them to show up 100%.” NT echoed these sentiments, remarking that “when people buy a product it has to sting a little bit if they purchase something, or else they won’t take it seriously and they won’t be fully invested.” Of course, given the four-figure prices that NT charges, this notion that it has to ‘sting’ to be useful may be somewhat justificatory. NT further rationalized his pricing approach by comparing his prices to the sums of money that many people with ME/CFS would spend on doctor’s visits, supplements, and other complementary and alternative medicines and services. Clearly, even though their entrepreneurship is positioned to move *away* from ‘careless’ medical markets, these markets continue to represent a point of comparison against which their own services were valued.

### Fostering Ethical Agency as a Motor Toward a Liveable Future

While the mainstream medical market represented one point of comparison, the second point, brought up throughout our interviews, was those therapists or alternative medical providers who created false hope: those who overpromised to patients, who stretched patients’ financial abilities to breaking point, and, worse, who held clients stuck in situations of dependency:

some [sufferers] are really going down a rabbit hole, spending so much money of stuff and getting themselves very stressed about it. Being told that they can’t get better unless they do … thousands, if not tens of thousands or hundreds of thousand dollars of treatments. (DM)

By contrast, our coaches explained how important it was to ‘dose’ the hope they gave to their clients: while opening up a new space of possibility and help trace a pathway toward this space, they emphasized the need to be clear that this pathway was an uncertain one, and that the work involved in following that pathway was substantial. ‘Dosing’ hope helped clients and coaches alike to avoid the trap of ‘cruel optimism’ (Berlant, 2012; see also Vidolov, Geiger & Stendahl, 2023) and instead imbue care with ethical agency as a motor toward the future. While some coaches stated that their goal was to guide people to complete recovery, therefore establishing radical hope and perhaps also a highly promissory future horizon for their clients, others were more cautious in their promises, careful not to feed clients’ magical thinking. PJ was particularly adamant that she saw her main role as helping people restore their agency in the here and now rather than promising a life of full health in some faraway future:

I describe myself almost as a mentor rather than a coach. They [clients] need somebody who understands, who gets them, and who is going to talk to them honestly and not promise them stuff that... Even if you were one of my clients and even if I magically knew everything that you needed to do to get better – I mean [this is] not possible, but say I knew every single thing – at the end of the day, it is down to you to do it. It is not my – I can’t fix you. (PJ)

Promises of full recovery were often also met with suspicion by potential clients who had had too many experiences of falling for ‘false hope’ and gaining no agency. On this basis, some sufferers rejected recovery coaching altogether, which they saw as yet another way of individualizing hope when the community really needed structural solutions that would restore agency to every sufferer—for instance in the form of social security benefits or reimbursement for medical treatments:

And then I found the other side of chronical illnesses. People say, “No, why are you saying this. This is chronic, this is forever, what we need is to get subsidised, we need this capacity, we need the monthly [social welfare] payments because this is very, very expensive, and you are telling us that it can be cured with some psychological programme”. (MV)

Evidently, the balance between hope that fosters ethical agency and false hope is a delicate one in these marketized contexts that hope’s sellers needed to tread lightly but that buyers also need to negotiate for themselves. As MV admits: “I think people need to understand that chronic doesn’t mean forever. Doesn’t mean no future. They have to find the balance.”

### A Moral Market for Hope?

The comment above, about structural supports for sufferers, leads us to the question of how far an individualized solution such as recovery coaching can go in building a moral market for people with chronic conditions such as ME/CFS. Accordingly, when we probed our research participants about their decision to become illness entrepreneurs, we also quizzed them about why they did not become more active in patient advocacy. Though some coaches maintained links with ME/CFS or Long Covid organizations, none of them perceived themselves as advocates. However, despite marketizing their services—and against conventional imageries of markets as arenas of competition—many of our participants described the market for recovery coaching as an ‘ecology’ of community support. In this ecology, coaches worked to collectively help as many people as possible, similar to the ‘ecology of care’ that Antoni and colleagues (2023) described in the French child protection agency they studied. Given that the people in our study were trying to make a living out of their coaching work, it was notable that none of them (not even NT with his aggressive business approach) considered the others as competitors. This was largely because they recognized that overall, there was not enough expertise to help every sufferer: “The world needs more, more, and more, and more [of us]” (PJ). PJ explained that she has a long waitlist of people who would like to work with her, and so having more coaches willing to do this work would benefit everyone. Similarly, TA described her “abundance mindset for success,” explaining that YouTube (where most of her work is based) and other easily accessible digital platforms were crucial tools to build a larger community of practice around ME/CFS recovery.

In contrast with most other research participants’ vision when asked about their own prospects, in keeping with his business mindset, NT articulated a vision of a market with ‘exciting’ growth, taking up a more familiar entrepreneurship idiom of market expansion:

there is no shortage of the audience. Especially with long Covid […] it is a growing market. In marketing, we would call this a blue ocean. Like there’s no bloodshed in the market. …. There’s probably not enough programs, if anything. So that’s where someone like myself comes in and builds a programme, we have got the skills. The next five years, I think this window of time is the time to grow something like this and help people, too. People are getting Covid, long Covid. So it’s exciting. (NT)

The apparent carelessness that percolates this quote is somewhat offset by his view of other coaches, indicating that “there is some kind of mutual respect because the intention is to help people” (NT). Like others in our sample, PJ leans toward a much more caring attitude, speaking with enthusiasm about one of her former clients who has decided to set out as a fatigue coach herself: “And now she is another support person for me. … now there is another fatigue coach in the UK who I can kind of use as a confidante as well, I guess” (PJ). TA offered perhaps the most realistic vision of the market that recovery coaches were building, underlining once again the continuous balancing act between making a hopeful living for oneself and serving the community:

I think it’s somewhere in the middle of working together but also a little bit of competitiveness. Which is a weird feeling when you are working with people, sick people. Like, no one wants to compete over this. But they are also people who have bills to pay and are trying to make a career and have a life, so it’s tough.

Overall, our interviews were shot through with a sense that what these recover coaches were hoping to do was indeed akin to building a moral market: a market that is different from mainstream medical markets both by offering hopeful pathways to its entrepreneurs and by contributing to an ecology of hope in the midst of a medical landscape that strips people of agency. We kept noting the inner struggles and conflicts most of our coaches had in determining the ‘edge’ between the two types of care—care toward self and other—and in fostering agency both for themselves and others, giving each party in the exchange an impetus to move toward a better future. The fact that they were reflective of these conflicts and continuously seeking to resolve them brought us to label this balancing act as ‘ethical agency,’ an agency that acts as the fuel to hope’s engine. In its broadest terms, one participant described this work and its inherent struggles as prefiguring hope as a foundation for a much broader ethical agency—namely a hopeful outlook for the whole of humanity:

I think that people with stuff like ME/CFS are a vanguard of a necessary, a wave of awakening and awareness about the sheer unsustainability of the way that we construct the human, the non-human, the way we even imagine the world – completely destructive and unsustainable. And that it’s fundamentally important to actually get back, or forwards, or all of it, to ontological, epistemological, and ethical commitments that actually do justice to what’s here, to who’s here in the entanglements that we are in and that we live as and that we live in.

## Discussion

Exploring illness entrepreneurs’ complex moral struggles around fostering and harnessing market-based hope not only serves to illuminate the complexities of ‘critical hope’ (Endrissat & Lüthy, 2025). It also highlights the role that hope plays in developing and enacting ethical agency. Extending and modifying Johansson and Wickström’s (2023) definition of ethical agency as care for others, we see it more akin to a continuous balancing act between self- and other-agency and as a motor for enacting hope on the basis of a caring disposition. Accordingly, we define ethical agency as agency that builds one’s own and others’ capacities and capabilities to overcome a painful present.

We draw two conceptual contributions from our deep engagement with our entrepreneurs of hope: first, we offer a new way of conceptualizing morality in markets, thinking through the morality of marketized care services as something that ought to include ethical agency, as defined in this paper. Second, we start contouring an ethics of hope, which might go some way toward building what Hudson et al. (2025) call an ‘architecture of hope’ in organization studies and business ethics, analyzing how care, hope, and ethical agency may work together to move people out of painful presences into new spaces of possibility.

### Hopeful Markets—Moral Markets?

Our findings highlight hope’s role in bringing into being a market for recovery coaches’ services and in keeping various market participants, both buyers and sellers, in the market’s fold. Hope acts as a complicated ethical practice in this market, intersecting its organization (how coaches frame the market exchange), its pricing (balancing livelihoods with providing access to supports), and the agency it fosters (in giving hope, but also bringing hopefulness into one’s own life). Throughout this research, we asked ourselves—and our research participants—the question of the ‘morality’ of this market work. As our findings demonstrated, coaches themselves struggled with this question: on the one hand, most are highly critical of the capitalist ‘rat race’ that causally contributes to the suffering they experienced and observed. On the other hand, they do not seek to challenge the economic inequalities that sit at the core of many people’s experiences with chronic illness, and rather than becoming patient advocates and activists, they have gravitated toward an individualizing market logic. This choice has consequences: the fact that they charge significant amounts of money for their services, that they have to allocate limited time for each client, that they spend their time entrepreneuring rather than fighting the system for collective resources and structural change. In this sense, our findings echo previous studies that have identified sites where care and market rationality make for awkward bed-fellows (e.g., Antoni, Reineke & Fotaki, 2020; Antoni, Reineke & Fotaki, 2023; Geiger, 2021; Geiger & Loza, 2024). At the same time, these coaches’ work is fuelled by the belief that by building an ecology of hope, they contribute to offering people an ethical alternative to what they had previously experienced. We described at length the continuous balancing acts that recovery coaches undertake in weighing up their own hopes for a livelihood with the good they think they can bring to the highest number of fellow sufferers possible. Questions of fair pricing and access were part of these struggles, as did the obligation to be emotionally available for their clients. While most (with one possible exception) did not consider their entrepreneuring as a way to get rich any time soon, ‘doing good’ and ‘doing well’ remained a continuous battle, which drew coaches in different directions.

Translating these insights into the entrepreneurship literature opens up a much deeper understanding of hope as a guardrail for ethical agency in certain types of entrepreneurship. This type of entrepreneurship is not necessarily ‘social’ in the conventional sense of the term (for instance, by combining a market and civic mission; see Martin et al., 2024) but it remains other-oriented and caring, stemming from shared experiences of pain, loss, and vulnerability. Literature in the sociology of health has amply described the biographical disruption caused by severe illness (Bury, 1982; Williams, 2000), and all of our research participants had experienced such a disruption. Rebuilding the pieces through entrepreneuring, they embodied hope for others, similar to O’Neill’s (2024) female wellness entrepreneurs who had experienced toxic relationships and who saw their entrepreneurship both as a way to escape from this suffering and to strengthen others’ resolves to do the same. In both cases, the ethical agency that results from overcoming such incisive life disruptions and rebuilding toward a liveable future is the real product in these markets: the possibility of emerging from pain and the modeling of new biographical beginnings. On this basis, illness and wellness entrepreneurs enact a specific form of moral market: a market that trades in hope not as cruel optimism but as an existential attitude and a pathway to action.

Of course, as with any moral market, it is vital not to naïvely romanticize the market’s practices; we do not exclude the possibility that those recovered from severe illness may turn into cut-throat and selfish rather than caring entrepreneurs (and we had some inkling of this in one of or research participants). Neither are we in a position to ascertain whether what these recovery coaches offered ultimately helped people any more than the products or services of other, less scrupulous merchants of hope (see Moriarty, 2024). In the final count, we have no way to determine whether the hope generated was not also ‘cruel’ (Berlant, 2012). We are further aware that hope in recovery is something that is highly personal and often also spiritual—hope, belief, and magical thinking are frequently inseparable—and that its marketization is almost always aimed at those who are already vulnerable. These are important ethical issues, complicating any claim as to the morality of the market we described in this paper and of the entrepreneurs building and maintaining it. More research is needed in other settings of markets of hope to put their morality to the test. At a minimum, our study corroborates the need for research to orient itself much more strongly toward entrepreneurs who are motivated by very different conceptions of the spaces of possibility that their entrepreneurship opens up, particularly in the space of illness, wellness, and lifestyle entrepreneurship (Ivanycheva et al., 2024): these entrepreneurs’ ethical agency lies in their quest to sketch temporal horizons where everyone may see themselves living liveable lives.

### Contouring an Ethics and Architecture of Hope

Conceptualizing our findings in the light of previous social theory, we propose that an ethics of hope may be defined as an ethical practice that enables individuals to develop their own goals, highlights pathways to fulfill these goals, and fosters agency for individuals to embark on these pathways. Seen in this light, our coaches’ hopeful work may indeed be understood as ethical practice: modeling to others ‘a life worth living’ (Alacovska, 2019) after illness, even if this life is cast within the market system. Moreover, the very fact that our coaches were highly reflective of the moral struggles posed by framing hope within a market logic may justify labelling this work as ethical practice. The deep, shared experience of suffering and vulnerability in turn framed how the coaches sought to resolve these struggles. This is reminiscent of Johansson and Wickström’s (2023) notion of ethical agency in ‘caring with’ others, a caring deeply anchored in shared sense of pain and vulnerability—but it is an ethical agency that is forward-looking, helping and encouraging people to move out of pain and toward new spaces of possibility, particularly if such spaces are enacted in a ‘future-in-the-present’ (Kleist & Jansen, 2016) rather than some faraway imaginary time.

While future research is required to contour an ethics of hope in more depth and detail, our findings may allow us to start answering Hudson et al.’s (2025) call for assembling the building blocks of an ‘architecture of hope’ in organizational and entrepreneurial life. Across our data, four such building blocks of hope emerged and interacted: pain/vulnerability, care, ethical agency, and spaces of possibility. Coaches described their caregiving in great detail and expanded at length on how they perceived their own work in direct contrast to the too-often careless and hope-less nature of mainstream medical markets. Yet, they also continually put this caring disposition in context with the temporality of hope—a hope that points toward a self-directed and self-determined life, opening up the possibility of leaving current pain and suffering behind. Recognizing the other through shared experiences of pain (Geiger et al., 2024; Johansson & Wickström, 2023; Vidolov et al., 2023; Wright 2023) fosters a focus on agency—both self and other. Clearly, care and hope stand in an intimate relationship to each other and may be difficult to disentangle practically; yet from our findings it seems clear that they might be at their most powerful when they are co-existent and aligned: care without hope can keep those receiving care in places of dependency and an ever-repeating present; similarly, markets for hope can be distinctly careless, such as in Perrotta and Hamper’s (2021) case of IVF treatment add-ons, where hope ‘sticks’ prospective parents to a future with a child but makes this future contingent on costly services.

We thus postulate that ethical agency requires both, care and hope—for self and others. Hope gives care the impetus to move people toward new spaces of possibility. In many contexts, hope points toward a horizon where pain is lessened; maybe even one where care is no longer needed or where those currently dependent on care may become care-givers themselves, just as our coaches have done. Hope cultivates an orientation that transcends and transforms the present moment and gives those enacting it a sense of ‘free action,’ to borrow an apt phrase from the Christian ethics of hope (Moltmann, 2012): the capacity and capability to move into the future. Ethical agency is the all-important fuel: the force that allows movement.  In turn, spaces of possibility provide the horizon to direct this movement toward.

In our particular case, the four vectors played into all facets of the moral struggles illustrated in our finding: how recovery coaches embodied and manifested hope, how they sought and achieved alignment, how they justified marketizing hope and putting a ‘price’ on it, how, despite this economic framing, they sought to foster agency in their clients, and how they built a more moral market of hope for a community all but abandoned by mainstream ‘care’ markets. Future empirical studies need to continue building out this architecture of hope in other cases and with other communities. Such studies should also continue the work started in this article of delineating the relationship between an ethics of care and an ethics of hope. These future studies should be particularly sensitive to the embodied and affective nature of hope, asking, with Anderson (2006, p. 734), “What can a body do when it becomes hopeful? What capacities, and capabilities, are enabled?” Indeed, perhaps our main task in tracing an ethics of hope is exactly this: ascertaining how different contexts may strengthen or diminish bodies’ capacities and capabilities to act—inside or outside of market structures.

## Data Availability

De-identified data is available from the authors upon request.

## Footnotes

1 This was a vital part of our research as those who stand outside of these illness experiences often find it extremely difficult to grasp what it is like living with ME/CFS.

2 This fact somewhat levels out, in our case, the otherwise significant differences between US and European-based healthcare provision. Usually, in the US, patients have to contend with much higher out-of-pocket payments than their European counterparts for most healthcare services.
